# Supplementary material for: Tree species identity and composition shape the epiphytic lichen community of structurally simple boreal forests over vast areas
Source: PLoS One. 2021 Sep 17;16(9):e0257564. doi: 10.1371/journal.pone.0257564 (PMC8448330; doi:10.1371/journal.pone.0257564)
Supplement: S1 File — (DOCX) [file pone.0257564.s001.docx]

**Tree species identity and composition shape the epiphytic lichen community of structurally simple boreal forests over vast areas**

Julian Klein, Matthew Low, Göran Thor, Jörgen Sjögren, Eva Lindberg, Sönke Eggers

Corresponding author:
Julian Klein, Swedish Species Information Centre, SLU, Uppsala, Sweden, julian.klein@slu.se

**Supplementary Information 1 - The hierarchical species accumulation model**

We based our hierarchical species accumulation (*hsac*) model on the Michaelis – Menten function (Tjørve, 2003), because its asymptote is the expected gamma diversity $\gamma$, and its half-saturation parameter *HS* is a measure of the among sample beta diversity (Kluth and Bruelheide, 2004). We here show that, with a minor modification of the Michaelis – Menten function, the half-saturation parameter can be replaced with true beta diversity *β* and the sample unit *u* at which alpha diversity *α* is evaluated (works for all units, e.g. discrete unit, area, volume, or time). First the original Michaelis – Menten function:

| $U_{i}=\frac{\gamma*i}{HS+i}$ | (1) |
| --- | --- |

When *i* = *u*, then *U_i_* = *α*, and because $\alpha=\frac{\gamma}{\beta}$, it follows:

| $\frac{\gamma}{\beta}=\frac{\gamma*u}{HS+u}$ | (2) |
| --- | --- |

Which solves for:

| $HS=\beta*u-u$ | (3) |
| --- | --- |

Which when substituted into equation 1, results in:

| $U_{i}=\frac{\gamma*i}{\beta*u-u+i}$ | (4) |
| --- | --- |

If the sampling process behind the species accumulation curve is discrete, then *u* = 1, and equation 4 is further simplified, as opposed to a continuous process as in species accumulation across areas for example. This model now allows for the simultaneous estimation of true beta and gamma diversity, which is ideally done using a hierarchical Bayesian approach. This because these methods enable us to fit equation 4 to the accumulation data and at the same time explain the equation’s parameters (true beta and gamma diversity) with any complex function through the addition of hierarchical levels on those parameters.

Kluth, C., Bruelheide, H., 2004. Using standardized sampling designs from population ecology to assess biodiversity patterns of therophyte vegetation across scales: Assessing biodiversity patterns across scales. J. Biogeogr. 31, 363–377. https://doi.org/10.1046/j.0305-0270.2003.00972.x

Tjørve, E., 2003. Shapes and functions of species-area curves: A review of possible models. J. Biogeogr. 30, 827–835. https://doi.org/10.1046/j.1365-2699.2003.00877.x
