# Supplementary material for: Tree species identity and composition shape the epiphytic lichen community of structurally simple boreal forests over vast areas
Source: PLoS One. 2021 Sep 17;16(9):e0257564. doi: 10.1371/journal.pone.0257564 (PMC8448330; doi:10.1371/journal.pone.0257564)
Supplement: S1 Table — Here species with a median probability of occurrence < 0.01 on all trees are shown. (DOCX) [file pone.0257564.s002.docx]

**Tree species identity and composition shape the epiphytic lichen community of structurally simple boreal forests over vast areas**

Julian Klein, Matthew Low, Göran Thor, Jörgen Sjögren, Eva Lindberg, Sönke Eggers

Corresponding author:
Julian Klein, Swedish Species Information Centre, SLU, Uppsala, Sweden, julian.klein@slu.se

| **Table S1. Epiphytic lichen species observed in this study and their probability of occurrence on a certain host tree species as well as on a tree in average**. Here species with a median probability of occurrence < 0.01 on all trees are shown. The numbers show the median and the lower and upper limit of the 95% credible intervals. The nomenclature follows Nordin et al. (2019). | | | | | | | |
| --- | --- | --- | --- | --- | --- | --- | --- |
| **Species identity** | **Alder** | **Aspen** | **Birch spp.** | **Oak** | **Pine** | **Spruce** | **Average tree** |
| *Absconditella lignicola* | 0(0-0.02) | 0(0-0.02) | 0(0-0.01) | 0(0-0.13) | 0(0-0) | 0(0-0) | 0(0-0.03) |
| *Arthonia vinosa* | 0(0-0.01) | 0(0-0.02) | 0(0-0) | 0(0-0.05) | 0(0-0) | 0(0-0) | 0(0-0.01) |
| *Bacidia beckhausii* | 0(0-0.02) | 0(0-0.02) | 0(0-0.01) | 0(0-0.09) | 0(0-0) | 0(0-0) | 0(0-0.02) |
| *Biatora helvola* | 0(0-0.01) | 0(0-0.02) | 0(0-0) | 0(0-0.05) | 0(0-0) | 0(0-0.01) | 0(0-0.01) |
| *Bryoria implexa* | 0(0-0.01) | 0(0-0.01) | 0(0-0) | 0(0-0.04) | 0(0-0) | 0(0-0.02) | 0(0-0.01) |
| *Calicium glaucellum* | 0(0-0.01) | 0(0-0.02) | 0(0-0) | 0(0-0.05) | 0(0-0) | 0(0-0) | 0(0-0.01) |
| *Calicium parvum* | 0(0-0.01) | 0(0-0.01) | 0(0-0) | 0(0-0.07) | 0(0-0.01) | 0(0-0) | 0(0-0.01) |
| *Catillaria erysiboides* | 0(0-0.01) | 0(0-0.02) | 0(0-0) | 0(0-0.06) | 0(0-0) | 0(0-0) | 0(0-0.01) |
| *Chaenotheca ferruginea* | 0(0-0.01) | 0(0-0.01) | 0(0-0) | 0(0-0.03) | 0(0-0) | 0(0-0) | 0(0-0.01) |
| *Chaenotheca furfuracea* | 0(0-0.01) | 0(0-0.02) | 0(0-0) | 0(0-0.07) | 0(0-0) | 0(0-0.01) | 0(0-0.02) |
| *Cladonia arbuscula* | 0(0-0.05) | 0(0-0.03) | 0(0-0.02) | 0(0-0.19) | 0(0-0.01) | 0(0-0) | 0(0-0.04) |
| *Cladonia botrytes* | 0(0-0.02) | 0(0-0.02) | 0(0-0.01) | 0(0-0.1) | 0(0-0) | 0(0-0) | 0(0-0.02) |
| *Cladonia deformis* | 0(0-0.03) | 0(0-0.02) | 0(0-0.01) | 0(0-0.12) | 0(0-0) | 0(0-0) | 0(0-0.02) |
| *Cladonia floerkeana* | 0(0-0.02) | 0(0-0.02) | 0(0-0.01) | 0(0-0.12) | 0(0-0) | 0(0-0) | 0(0-0.02) |
| *Cladonia macilenta* | 0(0-0.03) | 0(0-0.03) | 0(0-0.01) | 0(0-0.12) | 0(0-0.02) | 0(0-0) | 0(0-0.03) |
| *Cladonia rangiferina* | 0(0-0.04) | 0(0-0.03) | 0(0-0.02) | 0(0-0.17) | 0(0-0.01) | 0(0-0) | 0(0-0.03) |
| *Cladonia sulphurina* | 0(0-0.01) | 0(0-0.02) | 0(0-0) | 0(0-0.06) | 0(0-0) | 0(0-0) | 0(0-0.01) |
| *Fellhanera boutellei* | 0(0-0.01) | 0(0-0.01) | 0(0-0) | 0(0-0.05) | 0(0-0) | 0(0-0.01) | 0(0-0.01) |
| *Japewia subaurifera* | 0(0-0.02) | 0(0-0.02) | 0(0-0.01) | 0(0-0.09) | 0(0-0.01) | 0(0-0.01) | 0(0-0.02) |
| *Lecanora expallens* | 0(0-0.01) | 0(0-0.02) | 0(0-0) | 0(0-0.05) | 0(0-0) | 0(0-0) | 0(0-0.01) |
| *Lecidea erythrophaea* | 0(0-0.02) | 0(0-0.08) | 0(0-0.01) | 0(0-0.07) | 0(0-0) | 0(0-0) | 0(0-0.02) |
| *Loxospora elatina* | 0(0-0.01) | 0(0-0.02) | 0(0-0) | 0(0-0.04) | 0(0-0) | 0(0-0) | 0(0-0.01) |
| *Micarea lignaria* | 0(0-0.01) | 0(0-0.02) | 0(0-0) | 0(0-0.05) | 0(0-0) | 0(0-0) | 0(0-0.01) |
| *Micarea melaena* | 0(0-0.01) | 0(0-0.02) | 0(0-0) | 0(0-0.05) | 0(0-0) | 0(0-0) | 0(0-0.01) |
| *Micarea peliocarpa* | 0(0-0.01) | 0(0-0.01) | 0(0-0) | 0(0-0.04) | 0(0-0) | 0(0-0.01) | 0(0-0.01) |
| *Mycoblastus alpinus* | 0(0-0.06) | 0(0-0.02) | 0(0-0.01) | 0(0-0.08) | 0(0-0) | 0(0-0) | 0(0-0.02) |
| *Ochrolechia androgyna* | 0(0-0.04) | 0(0-0.08) | 0(0-0) | 0(0-0.07) | 0(0-0) | 0(0-0.01) | 0(0-0.03) |
| *Ochrolecia arborea* | 0(0-0.02) | 0(0-0.02) | 0(0-0.01) | 0(0-0.07) | 0(0-0) | 0(0-0.01) | 0(0-0.02) |
| *Pertusaria amara* | 0(0-0.02) | 0(0-0.02) | 0(0-0.01) | 0(0-0.1) | 0(0-0.01) | 0(0-0.01) | 0(0-0.02) |
| *Phaeophyscia orbicularis* | 0(0-0.01) | 0(0-0.09) | 0(0-0) | 0(0-0.03) | 0(0-0) | 0(0-0) | 0(0-0.02) |
| *Placynthiella icmalea* | 0(0-0.03) | 0(0-0.02) | 0(0-0.01) | 0(0-0.12) | 0(0-0.01) | 0(0-0) | 0(0-0.02) |
| *Polycauliona polycarpa* | 0(0-0.01) | 0(0-0.02) | 0(0-0) | 0(0-0.04) | 0(0-0) | 0(0-0.01) | 0(0-0.01) |
| *Pycnora sorophora* | 0(0-0.02) | 0(0-0.02) | 0(0-0.01) | 0(0-0.07) | 0(0-0) | 0(0-0.01) | 0(0-0.01) |
| *Ramalina farinacea* | 0(0-0) | 0(0-0.01) | 0(0-0) | 0(0-0.01) | 0(0-0) | 0(0-0.01) | 0(0-0) |
| *Scoliciosporum pruinosum* | 0(0-0.01) | 0(0-0.02) | 0(0-0) | 0(0-0.04) | 0(0-0) | 0(0-0.01) | 0(0-0.01) |
| *Scoliciosporum sarothamni* | 0(0-0.04) | 0(0-0.03) | 0(0-0.02) | 0(0-0.16) | 0(0-0) | 0(0-0) | 0(0-0.03) |
| *Stenocybe pullatula* | 0(0-0.05) | 0(0-0.02) | 0(0-0.01) | 0(0-0.09) | 0(0-0) | 0(0-0) | 0(0-0.02) |
| *Trapeliopsis granulosa* | 0(0-0.01) | 0(0-0.02) | 0(0-0) | 0(0-0.05) | 0(0-0) | 0(0-0) | 0(0-0.01) |
| *Xylopsora friesii* | 0(0-0.01) | 0(0-0.01) | 0(0-0) | 0(0-0.03) | 0(0-0) | 0(0-0) | 0(0-0.01) |

References:

Nordin, A., Moberg, R., Tönsberg, T., Vittikainen, O., Dalsätt, Å., Myrdal, M., Snitt-Ting, D., Ekman, S., 2019. Santesson’s checklist of Fennoscandian lichen-forming and lichenicolous fungi.
